# Submarine fresh groundwater discharge into Laizhou Bay comparable to the Yellow River flux

**DOI:** 10.1038/srep08814

**Published:** 2015-03-06

**Authors:** Xuejing Wang, Hailong Li, Jiu Jimmy Jiao, D. A. Barry, Ling Li, Xin Luo, Chaoyue Wang, Li Wan, Xusheng Wang, Xiaowei Jiang, Qian Ma, Wenjing Qu

**Affiliations:** 1 State Key Laboratory of Biogeology and Environmental Geology, China University of Geosciences, Beijing 100083, China; 2 MOE Key Laboratory of Groundwater Circulation & Environment Evolution and School of Water Resources and Environment, China University of Geosciences, Beijing 100083, China; 3 Department of Earth Sciences, The University of Hong Kong, Hong Kong, China; 4 Laboratoire de technologie écologique, Institut d'ingénierie de l'environnement, Faculté de l'environnement naturel, architectural et construit, Ecole Polytechnique Fédérale de Lausanne, Lausanne, 1015 Lausanne, Switzerland; 5 School of Civil Engineering, University of Queensland, Brisbane, Queensland; 6 State Key Laboratory of Hydrology-Water Resources and Hydraulic Engineering, Hohai University, Nanjing, China

## Abstract

Near- and off-shore fresh groundwater resources become increasingly important with the social and economic development in coastal areas. Although large scale (hundreds of km) submarine groundwater discharge (SGD) to the ocean has been shown to be of the same magnitude order as river discharge, submarine fresh groundwater discharge (SFGD) with magnitude comparable to large river discharge is never reported. Here, we proposed a method coupling mass-balance models of water, salt and radium isotopes based on field data of ^223^Ra, ^226^Ra and salinity to estimate the SFGD, SGD. By applying the method in Laizhou Bay (a water area of ~6000 km^2^), we showed that the SFGD and SGD are 0.57 ~ 0.88 times and 7.35 ~ 8.57 times the annual Yellow River flux in August 2012, respectively. The estimate of SFGD ranges from 4.12 × 10^7^ m^3^/d to 6.36 × 10^7^ m^3^/d, while SGD ranges from 5.32 × 10^8^ m^3^/d to 6.20 × 10^8^ m^3^/d. The proportion of the Yellow River input into Laizhou Bay was less than 14% of the total in August 2012. Our method can be used to estimate SFGD in various coastal waters.

Submarine groundwater discharge (SGD) is any and all flows of water on continental margins from the seabed to the coastal ocean, regardless of fluid composition or driving force^1^. SGD, which is driven by both terrestrial and marine forcing components, comprises terrestrial fresh groundwater (SFGD) and re-circulated seawater (RSGD)^1^,^2^. As an essential part of SGD, SFGD is an important source of freshwater, nutrients, contaminants, and other chemicals to the coastal waters, and has significant impacts and implications on coastal environment and ecology^3^,^4^. With near half of the global population residing within 100 km of the coastline^5^, it is important to find more freshwater resources including near- and off-shore fresh groundwater for the relief of water scarcity in densely populated coastal megacities^6^. Many previous studies have found that regional scale SGD is several times greater than river discharge^7^,^8^,^9^. For example, Kim et al.^7^ estimated the magnitude of SGD into the Yellow Sea to be at most 300% of the river water input. Moore et al.^8^ showed that the SGD flux is probably between 0.8 and 1.6 times the river flux to the Atlantic. Moore^9^ reevaluated the SGD to a large section of the South Atlantic Bight and found the annual average SGD flux is three times greater than the river fluxes. However, there are few studies on large scale SFGD as compared with those on SGD, and SFGD with magnitude comparable to large river discharge is never reported. In this paper, we proposed a method for combining mass-balance models of water, salt and radium isotopes to estimate the SFGD, SGD. By applying the method in Laizhou Bay, the SFGD, SGD, and the proportion of the Yellow River input into Laizhou Bay were estimated and discussed.

Laizhou Bay, located between 37.05°N ~ 37.80°N and 118.9°E ~ 120.35°E (WGS84 reference system), is one of the three major bays in the Bohai Sea, China (Fig. 1). The natural coastline of the Bay is relatively straight and extends from the Qimu Cape to the Yellow River Estuary. Along the coastline, there are at least four different types of depositional environments: the Yellow River delta in the northwest, the alluvial plain in the southwest, the marine deposit plain in the south, and the hilly area in the east^10^,^11^,^12^. The coast areas west of Hutouya (Fig. 1) are alluvial or marine deposit plains with aquifers mainly composed of permeable coarse material. The coast area east of Hutouya is hilly with a coastal plain. Groundwater occurs mainly in the Quaternary aquifers of the coastal plains. The average annual rainfall is ~640 mm and occurs mostly from June to September, while the potential evaporation is approximately 2050 mm^13^. The seawater depth of the Bay generally increases as the offshore distance increases, and has an average depth of ~8 m. Based on salinity measurements at surface, middle and bottom at the stations S2–S6 shown in Fig. 1, where the seawater is deepest in whole Laizhou Bay, it was found that the Bay is vertically well mixed and can be treated as a single layer in terms of the large-scale water motion.

There are many rivers flowing into the Bay, including the Yellow River, Jiaolai River, Xiaoqing River and Wei River. Particularly, the Yellow River, as the second and sixth largest river in China and the world, respectively, is the largest that discharges into the Bohai Sea. However, the Yellow River discharge has significantly decreased since the 1950s due to both climate change and human activities^14^,^15^. Seasonal variations of the Yellow River discharge are also significant, with the minimum occurring usually in April and May, and the maximum occurring usually in July and August (Fig. 2). The annual average discharge of the Yellow River in 2012 was 7.23 × 10^7^ m^3^/d. In July and August 2012, its discharge rate was at least 50 times greater than the sum of the discharge rates of all other rivers flowing into Laizhou Bay.

The Yellow River deposits large amounts of sediments, creating a fast-growing delta area. Its course into the Bohai Sea changed a number of times over the past several decades^16^. The present channel through the delta was formed artificially in 1996. The mouth of the Yellow River is located between Bohai Bay and Laizhou Bay, and the shifting of the mouth to the north may significantly reduce its direct discharge into Laizhou Bay.

As one of the three bays in the Bohai Sea, China, and with a shoreline of ~320 km and area of ~6000 km^2^, Laizhou Bay is an important coastal environment. As well, it is subject to a variety of environmental stresses, and hence provides an archetype of a semi-enclosed bay for which ecological functioning is a sensitive issue. Most existing studies on SGD in the Bohai Sea are restricted to the Yellow River delta^17^,^18^,^19^,^20^,^21^, the largest estuary in the Bohai Sea. In order to estimate SFGD, SGD, and the Yellow River input into Laizhou Bay, activities of radium isotopes (^223, 226^Ra) and salinities were measured (Fig. 1). Natural radium isotopes are ideal tracers for effective and efficient assessment of SGD since they are conservative chemically and widely enriched in groundwater relative to surface waters^7^,^8^,^21^,^22^. In August 2012, we collected eight groundwater samples, six river water samples, and 44 seawater samples for measurement of radium isotopes (^223, 226^Ra) and salinity (Supplementary Table S1). In May 2014, we measured the salinities at the same locations.

## Results

### Spatial distribution of radium isotopes and salinity

Figure 3 shows the spatial distributions of the two radium isotopes (^223, 226^Ra) and salinity within Laizhou Bay. The activity distributions of these two isotopes have the following common features: (1) the activities were significantly higher in the west and south than in the east and north of the Bay; and (2) the activities were very high in the estuary and near-shore areas and they generally decreased with the offshore distance (Figs. 3a and 3b). The seawater salinities in August 2012 were higher in the east than in the west of the Bay (Fig. 3c), where the activities of the radium isotopes were comparatively high. The seawater salinities in May 2014 were similar to those in August 2012 overall, but with a local low-salinity area near the middle of the eastern coast (Fig. 3d), indicating considerable SFGD since there are no river inputs in that region.

### Determination of flushing time

To explore the dynamics of coastal water, one should first estimate the flushing time *T_f_* [T] for a bay, i.e., the ratio of the mass or volume of a constituent (*V*) to its renewal rate (*Q*)^23^, or *T_f_* *=* *V*/*Q*. Combining the radium isotope method^24^ and the tidal prism method^25^, we obtained the following equation to estimate the flushing time *T_f_* based on measurements of radium isotopes,
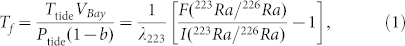
where *F*(^223^Ra/^226^Ra) is the^223^Ra/^226^Ra activity ratio of the flux into the bay, *I*(^223^Ra/^226^Ra) is the ^223^Ra/^226^Ra activity ratio in the bay, and *λ*_223_ = 0.061d^−1 ^is the decay constant of ^223^Ra. Using equation (1), we estimated *T_f_* *= * 36.6 ± 5.3 d, which is in line with previous independent estimates^17^,^20^,^26^ (see Flushing Time Model in Supplementary Information).

### Assessment of SFGD and SGD

To quantify the freshwater fluxes into Laizhou Bay, we developed water-mass and salt-mass balance models that include seawater from outside the Bay, river input, SFGD, precipitation (*P_T_*) and evaporation (*E_T_*), all estimated over the period of *T_f_* d immediately before the observation time (taken as the mid-point of the 8-d field sampling period).

Coupling the salt-mass balance model with the water-mass balance model, we derived the SFGD flux (*Q_SFGD_*) as (see Water and Salt Mass Balance Model in Supplementary Information):
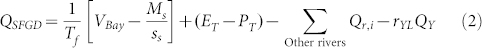
where *r_YL_* is the ratio of the Yellow River input into Laizhou Bay to the total input into the Bohai Sea (*Q_Y_*) during the flushing time immediately before the observation time; *Q_r,i_* is runoff of the *i*^th^ river other than the Yellow River into the Bay (Supplementary Table S2); *M_s_* is the total salt mass in the Bay; and *S_s_* is the salinity of the representative seawater outside of the Bay. Detailed calculations of these parameter values are described and summarized in Supplementary Table S3.

In order to quantify the fluxes of SGD into Laizhou Bay, we developed a ^226^Ra mass balance model within the whole bay. The inputs of ^226^Ra are from discharge waters (river, SGD, and seawater input from the open Bohai Sea outside of the Bay), desorption from suspended particles and diffusion from bottom sediments. Neglecting decay, gain from precipitation and loss from evaporation, the loss of ^226^Ra only includes mixing with the open sea. Using the steady state premise, one can ignore the variation of ^226^Ra storage in the Bay. In this case the SGD flux is given by (see ^226^Ra Mass Balance Model in Supplementary Information)
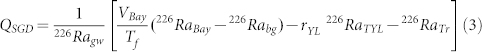
where ^226^*Ra_TYL_*is the total input of ^226^Ra from the Yellow River discharge and suspended particle desorption; ^226^*Ra_Tr_*is the total input of ^226^Ra from discharges and suspended particle desorption of all rivers other than the Yellow River; ^226^*Ra_gw_* and ^226^*Ra_Bay_* are the radium activity in groundwater and Bay water, respectively; and ^226^*Ra_bg_* is the background activity. Detailed calculations of these parameter values are described and summarized in Supplementary Table S4.

Figure 4a shows how the SFGD and SGD fluxes change with the proportion of the Yellow River input into Laizhou Bay (*r_YL_*). The annual average flux *Q*_Y2012_ = 7.23 × 10^7^ m^3^/d of the Yellow River in 2012 was used as the reference, which is more than 20 times the fluxes of all the other rivers flowing into Laizhou Bay. One can see that both SFGD and SGD decrease as *r_YL_* increases. Note that SGD is much greater than SFGD since SGD includes RSGD. From Fig. 4a, since SFGD ≥ 0, we have SGD ≥ 5.13*Q*_Y2012_ and *r_YL_* *≤* 0.396. When *r_YL_* = 0, we obtain the upper-bound estimate of SGD as 8.57*Q*_Y2012_ and the corresponding value of SFGD as 0.88*Q*_Y2012_. In general, we have SGD = (8.57 − 8.69*r_YL_*)*Q*_Y2012_, and SFGD = (0.88 − 2.22*r_YL_*)*Q*_Y2012_.

Seasonal variations of the Yellow River flux were significant. The minimum flux occurred in April–May and was less than one-tenth of the maximum flux in July–August (Fig. 2) during 2012 and 2013. Thus, SFGD estimation based on equation (2) using the flux of the Yellow River during April–May can effectively reduce the impact of the Yellow River. Considering that tides are the dominant forcing for the renewal of the seawater in the Bay^27^, it is reasonable to assume that the seasonal variation of the flushing time in Laizhou Bay is negligible. So the flushing time of *T_f_* *= * 36.6 ± 5.3 d, which was obtained with radium data in August 2012, may also be used for April–May 2014. Figure 4b shows how the SFGD predicted by equation (2) using the salinity data observed in May 2014 changes with *r_YL_*, the proportion of the Yellow River input into Laizhou Bay, during April and May in 2014. As *r_YL_* increases from 0 to 1, the SFGD decreases from 0.79*Q*_Y2012_ to 0.34*Q*_Y2012_. As expected, the impact of the Yellow River flux on the SFGD was significantly reduced by comparison with that of August 2012 (Fig. 4a).

The precipitation in Laizhou Bay is much larger in June–August than in March–May, and so the groundwater table in the coastal area should be higher (i.e., higher hydraulic head) in August than in May owing to rainfall infiltration into ground surface. Due to the increased landward gradient in hydraulic head, it follows that SFGD (August 2012) ≥ SFGD (May 2014). Using the lower-bound estimate for SFGD in May 2014 (0.34*Q*_Y2012_), from Fig. 4a one can see that if SFGD in August 2012 ≥ 0.34*Q*_Y2012_, then *r_YL_* (August 2012) was less than 0.24 and SGD (August 2012) ≥6.48*Q*_Y2012_ (as is indicated by the thick red-dashed vertical line in Fig. 4a).

Although we do not have direct evidence to conclude that *r_YL_* (May 2014) ≤0.24 since *r_YL_* varies with time, it should be considerably less than unity. In addition, the Yellow River mouth, which is located between Bohai Bay and Laizhou Bay, is northerly-oriented, as shown in photographs taken by NASA Landsat in May 2014 or Fig. 2 of Xu et al.^21^. This fact may significantly limit the input of the Yellow River into Laizhou Bay. Thus, it is at least not unreasonable to assume *r_YL_* (May 2014) ≤0.50. From Fig. 4b one can see that *r_YL_* (May 2014) ≤0.50 implies SFGD (May 2014) ≥0.57*Q*_Y2012_. Since SFGD (August 2012) ≥SFGD (May 2014), from Fig. 4a one can see that SFGD in August 2012 was in the range (0.57 ~ 0.88)*Q*_Y2012_, and *r_YL_* (August 2012) was less than 0.14 and SGD in August 2012 was between 7.35*Q*_Y2012_ (as is indicated by the thin red-dashed vertical line in Fig. 4a) and 8.57*Q*_Y2012_.

The seawater circulation in the Bohai Sea shows apparent seasonal variations, which in turn affect the path of the Yellow River discharge^28^. During the summer months, the monsoonal winds blow from the south in this region, thus creating a cyclonic gyre within the Bohai Sea^26^,^28^. These may limit the input of the Yellow River into Laizhou Bay and support the small estimated value of *r_YL_* (August 2012).

## Discussion

Our tracers-orientated estimation of SGD is approximately ten times as large as SFGD. SGD is much greater than SFGD since SGD = SFGD + RSGD, and many previous studies show that tides, waves, fluid density gradient, storms, geothermal gradient, and seabed topography can result in RSGD^29^,^30^,^31^,^32^,^33^,^34^,^35^,^36^. As a result, the SGD values estimated by the tracers-orientated method and mechanism-orientated method such as traditional hydrogeological approach often do not match. Li and Jiao^29^ reviewed the studies of tidal contribution to SGD and found that the RSGD induced by tides in the intertidal zone is at least 1 ~ 2 orders of magnitude smaller than SGD estimated by radium isotope tracers. The order of magnitude of RSGD contributed by waves is the same as that of tides^30^,^31^. The density-driven RSGD is usually much less than SFGD^32^,^33^,^34^. The RSGD induced by geothermal gradient and seabed topography is at least 4 orders of magnitude smaller than the SGD estimated by radium isotope tracers^35^,^36^,^37^. Thus, SGD estimated by radium isotope tracers is much larger than that of the total sum of SGD induced by all the above factors/mechanisms. Solving this challenging problem needs long-term efforts involving close combination of tracers-orientated and mechanism-orientated methods.

We can compare our results with small-scale SGD studies. Taniguchi et al.^19^ used seepage meters to estimate SGD fluxes of 2300 m^3^/m/d in September 2004, 3065 m^3^/m/d in September 2006 for a 7-km offshore area approximately 40 km south of the Yellow River estuary. They also estimated the average SFGD of 18 and 28 m^3^/m/d in September 2004 and 2006, respectively. Based on their results, the average ratio of SFGD to SGD is less than 1% in both sampling periods. Our results, if using the same unit (treating SGD as a line source along the coastline and dividing the total SGD by the shoreline length of the Bay), ranges from 1662.2 m^3^/m/d to 1937.2 m^3^/m/d, which are slightly smaller than Taniguchi's estimates. The ratio of SFGD to SGD in our study, ranging from 7.5% to 9% as shown in Fig. 4a when *r_YL_* ≤ 0.14, however, is much higher than that of their study. Peterson et al.^18^ estimated SGD in the same area as Taniguchi et al.^19^ using radon and radium isotopes. They estimated a SGD flux of 4.5–13.9 cm/d in September 2006, most of which was recirculated seawater. Our results, if using the same unit (treating SGD as an area source and dividing the total SGD by the area of the Bay), ranges from 8.9 cm/d to 10.3 cm/d, which are within the range of Peterson's estimates for the same season. Since our tracers-orientated estimations of SGD and SFGD are based on the mass balance of radium isotopes and fresh groundwater after they entered the seawater, they are conservative in the sense that if there had no groundwater exploitations in the coastal area of Laizhou Bay^12^,^38^, their values would be larger.

**Figure 1:**
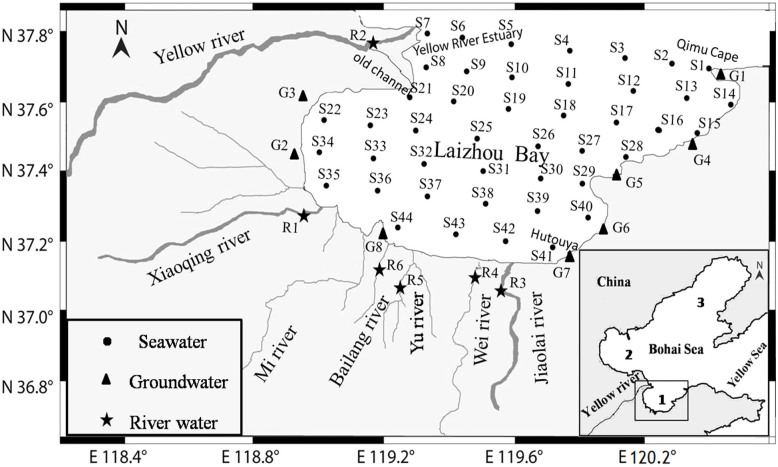
Laizhou Bay and sampling stations. The dots, triangles and pentagrams represent sampling stations for seawater (S), groundwater (G) and river water (R), respectively. Station numbers as defined in Supplementary Table S1 are marked next to each of the stations. The numbers 1, 2, and 3 in the inset indicate Laizhou Bay, Bohai Bay and Liaodong Bay, respectively. Maps were created with MAPGIS 6.7 software.

**Figure 2:**
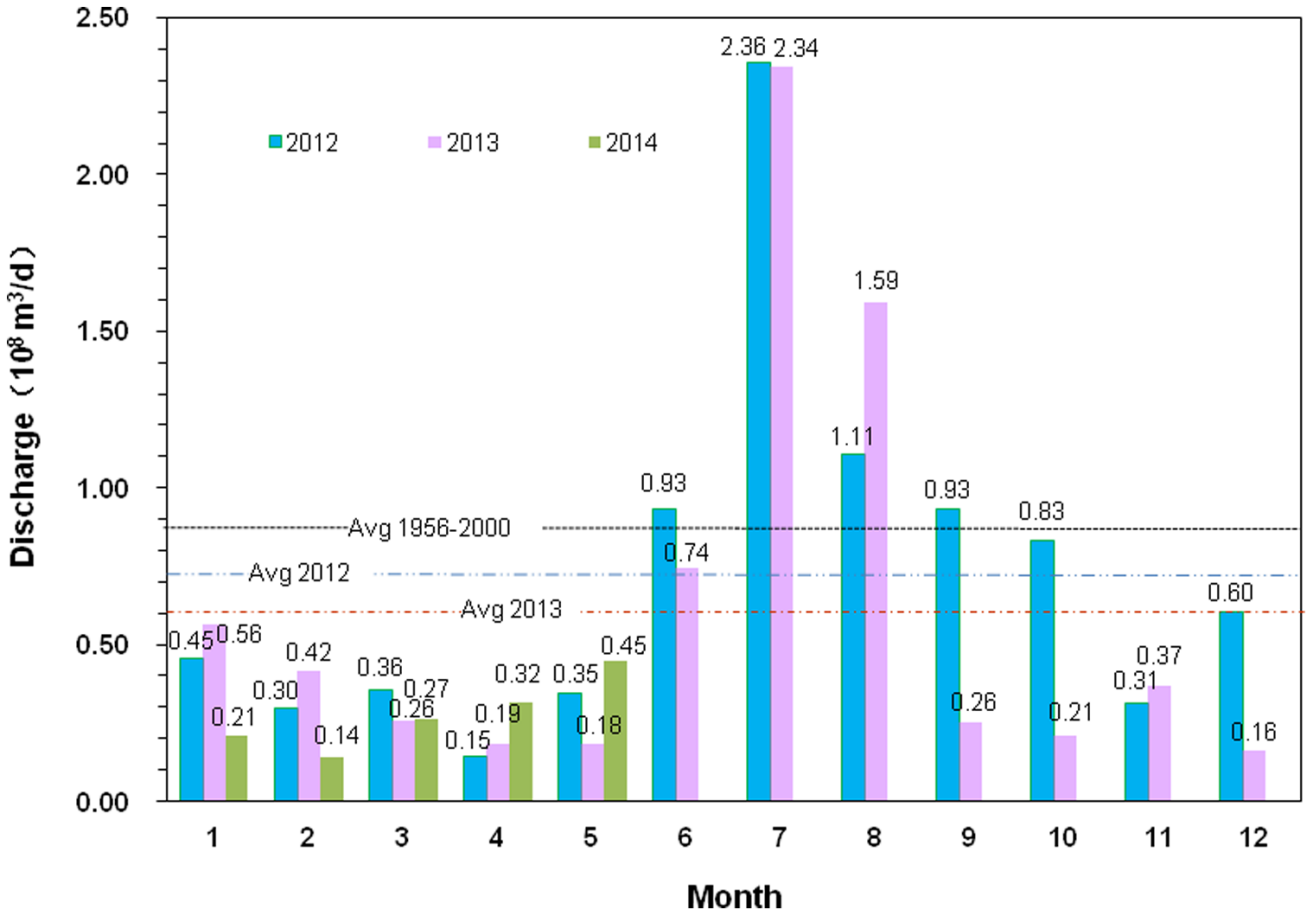
Monthly average water discharge of the Yellow River to the Bohai Sea from 2012 to 2014. The dashed black, blue and red lines represent annual average discharge from 1956 to 2000, in 2012, and 2013, respectively. Data from the Yellow River Water Resources Bulletin of Yellow River Conservancy Commission of the Ministry of Water Resources.

**Figure 3:**
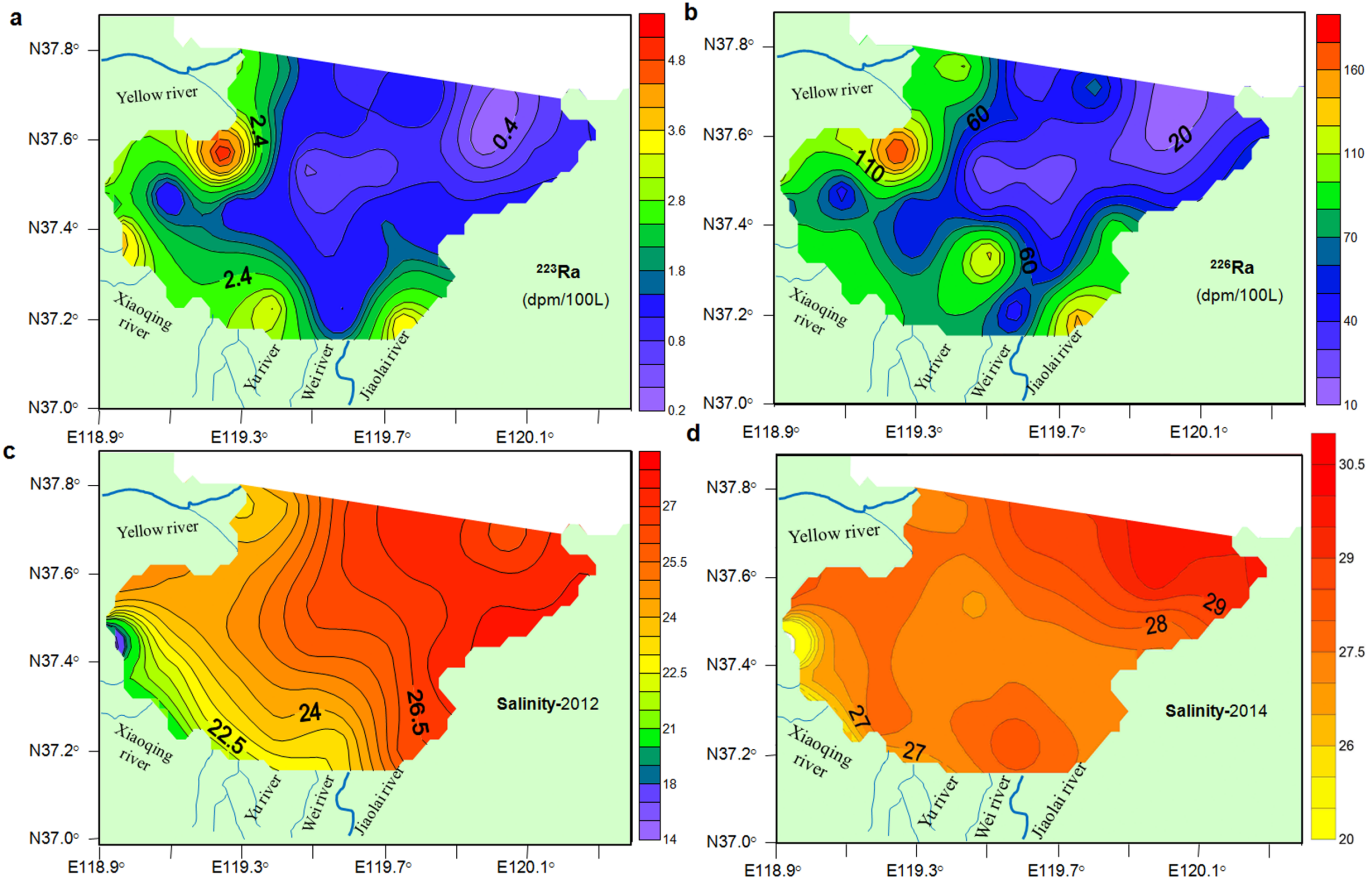
Contour plots. (a), ^223^Ra in August 2012. (b), ^226^Ra in August 2012. (c), Salinity in August 2012. (d), Salinity in May 2014. Activities of radium isotopes and salinity were measured in surface water (1–2 m below the surface) of Laizhou Bay from 19 to 26 August 2012. Salinity was also measured at the same locations from 1 to 6 May 2014. Maps were created with Surfer 8.0 software.

**Figure 4:**
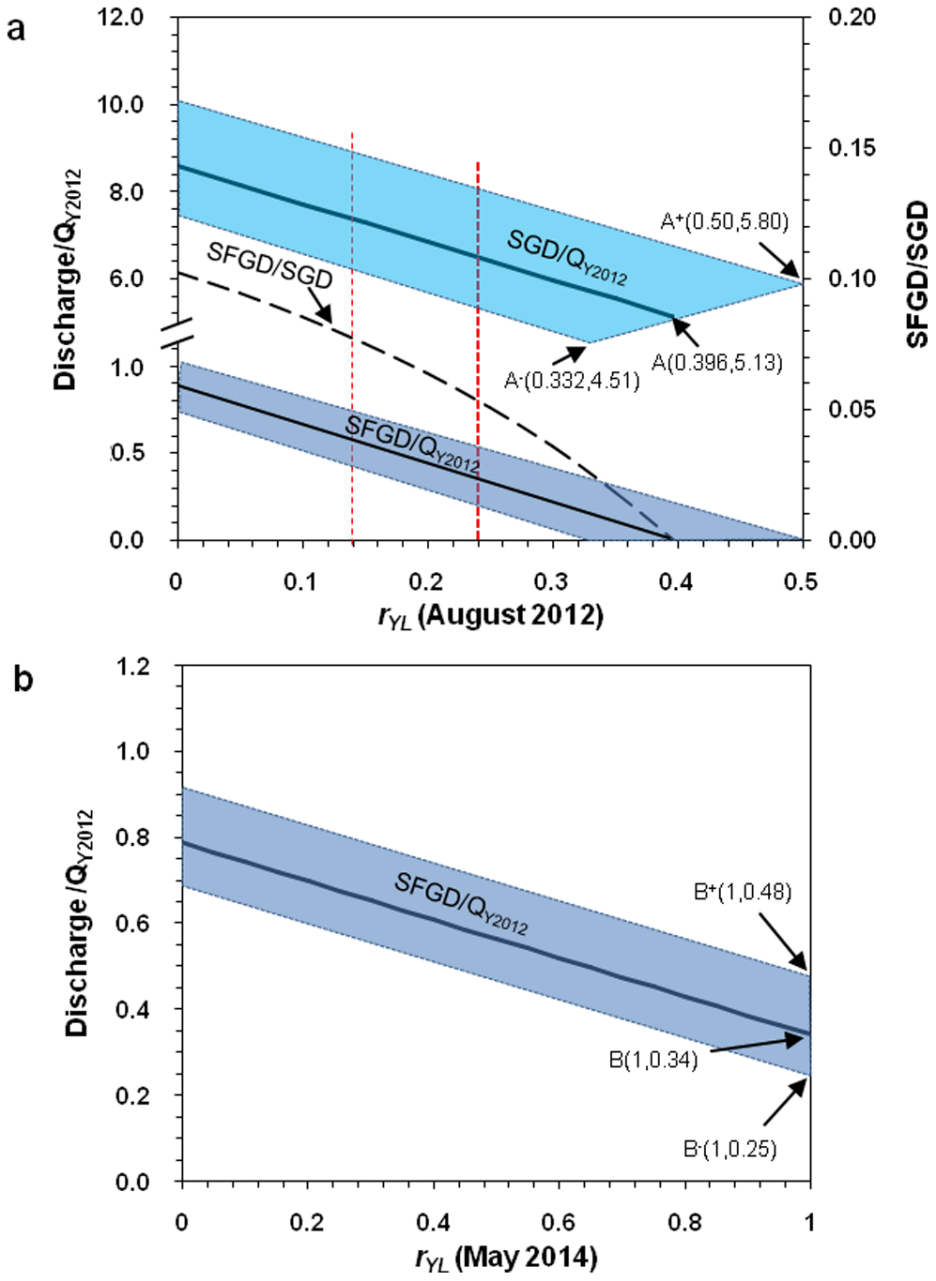
Changes of SFGD and SGD with the proportion of Yellow River input into Laizhou Bay. The SFGD and SGD are nondimensionalized by *Q*_Y2012_, the annual average flow rate of the Yellow River in 2012, which equals 7.23 × 10^7^ m^3^/d. (a), SFGD and SGD as predicted by equations (2) and (3) using data from August 2012. The ratio of SFGD to SGD is shown on the right-axis. (b), SFGD as predicted by equation (2) using salinity data in May 2014. The blue bands bounded by the pair of dotted-lines represent the error ranges corresponding to the range of *T_f_* = 36.6 ± 5.3 d, with longer flushing time corresponding to smaller SFGD or SGD. Note that the parameter *r_YL_* varies with time.

The Bohai Sea has three major bays: Laizhou Bay, Bohai Bay and Liaodong Bay (Fig. 1). The seawater salinity in Laizhou Bay was significantly lower than that in other two bays^39^. The real reason for this was not investigated in detail because it seems consistent with the freshwater discharge from the Yellow River abutting Laizhou Bay. Our investigation gives a plausible explanation for the abnormally low salinity in Laizhou Bay. We concluded that the SFGD, which accounts for 57% ~ 88% of the annual flux of the Yellow River in 2012, is a key contributor to the low salinity given that the direct input from the Yellow River to Laizhou Bay is very limited (*r_YL_* ≤ 14% in August 2012).

SGD has been widely recognized to be a pathway for enriching coastal waters in nutrients, carbon and metals^1^,^8^,^22^. In some areas, nutrient fluxes via SGD were shown to rival those from surface waters^7^,^40^,^41^. The nutrient input via SGD in the Yellow River Estuary is at least five times of that via the Yellow River^20^. With new understanding of our assessments of the SFGD, SGD and the Yellow River input into Laizhou Bay, the management of the Bay related to fresh groundwater resources, ecology and environment in coastal and offshore areas should be reviewed.

Although SGD has been estimated in many coastal areas all over the world based on radium isotope tracer methods^29^,^42^, large scale tracers-orientated SFGD studies have not been correspondingly conducted even if SFGD is as important as, or even more important than SGD. In this study, we proposed a tracers-orientated method (using radium isotopes and salinity in seawater and coastal groundwater as tracers) to estimate SFGD. To the knowledge of the authors, this is the first time to quantify large-scale SFGD using a tracers-orientated method based on field radium and salinity measurements. Our method has potential for application considering that the seawater salinity is a common physical quantity that can be measured easily and a straightforward tracer to indicate freshwater. The proposed method can be readily applied to estimate SFGD in other coastal areas all over the world.

The main limitation of current study is most probably the steady state premise, a common approach used in all the previous tracers-orientated SGD studies by radium isotope methods^7^,^8^,^22^,^24^,^43^. Since the variation of radium storage in the whole bay approaches zero near the time when the radium mass in the whole bay reaches its maximum, and the observation period of our field work is just near such a maximum-time, the steady state assumption is approximately valid. Strict quantification of the error induced by steady state assumption, however, needs not only much more radium isotope measurement data in terms of time series, but also quantification of the seawater flow in the whole bay for a long period. This will be an interesting and challenging work in the future.

## Methods

### Sampling

Radium samples were collected from and adjacent to Laizhou Bay. Large volume water samples (~60 L for seawater, ~15 L for river water, respectively) were pumped and filtered through a 0.45-μm filter for Ra extraction. The coastal groundwater samples (~15 L) were taken from the nearshore zone within 100 m of the high tide mark with PushPoint samplers inserted into sediments at a depth of ~1.5 m. These samples can capture the chemical components (particularly radium isotopes) of groundwater immediately before it discharges into the sea from the aquifer. The water samples were passed slowly through Mn-fibers (~25 g) produced according to the method proposed by Moore^44^ for extracting radium isotopes. The flow rate was controlled not to exceed 1 L/min to ensure complete Ra adsorption on the Mn-fiber. These fibers were then washed thoroughly to remove all particles and salts and taken to the hydrogeological laboratory at The University of Hong Kong for measurements. The salinity, temperature, pH of the water samples were measured in situ using a HI9828 Model probe (HANNA).

### Measurements

The long-lived radium isotope, ^226^Ra, was determined by a radon-in-air monitor (RAD7, Durridge Co.) as proposed by Kim et al.^45^. After the ^223^Ra measurements were completed, the Mn-fiber samples were aged for 2 - 6 weeks to allow ^222^Rn and its daughters to equilibrate with ^226^Ra. This is an indirect measurement of ^226^Ra based on secular equilibrium between ^226^Ra and ^222^Rn. In order to improve the test results, the determination of ^226^Ra in our study slightly modified the method of Kim et al.^45^. Before measurement we made long fiber incubation time (> 20 d) to ensure secular equilibrium between ^226^Ra and ^222^Rn and then allowed ~24 h for purging the RAD7 system prior to analysis thereby reducing the background noise. In addition, we reduced errors by using long measurement times^46^. The expected error of ^226^Ra measurements is ±7%. The short-lived radium isotope ^223^Ra was analyzed using a two-channel radium delayed coincidence counting system (RaDeCC)^47^. The expected error of ^223^Ra measurements is ±12%.

### Models

Three models (flushing time, water and salt mass balance models) as well as the ^226^Ra mass balance model were used in the study to estimate the flushing time of coastal water, the freshwater discharge, the Yellow River input, and SGD, respectively. Details were shown in Supplementary Information.

## Supplementary Material

Supplementary Information Supplementary Information

## References

[b1] Burnett W. C., Bokuniewicz H., Huettel M., Moore W. S. & Taniguchi M. Groundwater and pore water inputs to the coastal zone. Biogeochemistry 66, 3–33, 10.1023/b:biog.0000006066.21240.53 (2003).

[b2] Moore W. S. The effect of submarine groundwater discharge on the ocean. Annu. Rev. Mar. Sci. 2, 59–88, 10.1146/annurev-marine-120308-081019 (2010). 21141658

[b3] Schmidt A., Santos I. R., Burnett W. C., Niencheski F. & Knöller K. Groundwater sources in a permeable coastal barrier: Evidence from stable isotopes. J. Hydrol. 406, 66–72, 10.1016/j.jhydrol.2011.06.001 (2011).

[b4] Slomp C. P. & Van Cappellen P. Nutrient inputs to the coastal ocean through submarine groundwater discharge: controls and potential impact. J. Hydrol. 295, 64–86, 10.1016/j.jhydrol.2004.02.018 (2004).

[b5] Martínez M. L. *et al.* The coasts of our world: Ecological, economic and social importance. Ecol. Econ. 63, 254–272, 10.1016/j.ecolecon.2006.10.022 (2007).

[b6] Post V. E. A. *et al.* Offshore fresh groundwater reserves as a global phenomenon. Nature 504, 71–78, 10.1038/nature12858 (2013). 24305150

[b7] Kim G., Ryu J. W., Yang H. S. & Yun S. T. Submarine groundwater discharge (SGD) into the Yellow Sea revealed by Ra-228 and Ra-226 isotopes: Implications for global silicate fluxes. Earth. Planet. Sc. Lett. 237, 156–166, 10.1016/j.epsl.2005.06.011 (2005).

[b8] Moore W. S., Sarmiento J. L. & Key R. M. Submarine groundwater discharge revealed by Ra-228 distribution in the upper Atlantic Ocean. Nat. Geosci. 1, 309–311, 10.1038/ngeo183 (2008).

[b9] Moore W. S. A reevaluation of submarine groundwater discharge along the southeastern coast of North America. Global Biogeochem. Cy 24, GB4005, 10.1029/2009GB003747 (2010).

[b10] Han C. R., Tan Q. X., Jiang Y. C., Sun Y. & Liu Q. R. Quaternary sedimentation and paleogeographic characteristics of littoral area in the east of the Laizhou Bay. Mar. Geol. & Quaternary Geol. 16, 75–84 (1996). (in Chinese)

[b11] Li M., Wang Q., Zhang A. & Liu Y. Study on the geomorphic evolution of the muddy coast along the southern-western Laizhou Bay over the past 50 years. Mar. Sci. Bull. 32, 141–151 (2013) (in Chinese).

[b12] Han D. M., Song X. F., Currell M. J., Yang J. L. & Xiao G. Q. Chemical and isotopic constraints on evolution of groundwater salinization in the coastal plain aquifer of Laizhou Bay, China. J. Hydrol. 508, 12–27, 10.1016/j.jhydrol.2013.10.040 (2014).

[b13] Chen J. *et al.* Nitrate pollution of groundwater in the Yellow River delta, China. Hydrogeol. J. 15, 1605–1614, 10.1007/s10040-007-0196-7 (2007).

[b14] Wang H., Yang Z., Saito Y., Liu J. P. & Sun X. Interannual and seasonal variation of the Huanghe (Yellow River) water discharge over the past 50 years: Connections to impacts from ENSO events and dams. Global Planet. Change 50, 212–225, 10.1016/j.gloplacha.2006.01.005 (2006).

[b15] Wang H. *et al.* Stepwise decreases of the Huanghe (Yellow River) sediment load (1950–2005): Impacts of climate change and human activities. Global Planet. Change 57, 331–354, 10.1016/j.gloplacha.2007.01.003 (2007).

[b16] Chu Z. X., Sun X. G., Zhai S. K. & Xu K. H. Changing pattern of accretion/erosion of the modem Yellow River (Huanghe) subaerial delta, China: Based on remote sensing images. Mar. Geol. 227, 13–30, 10.1016/j.margeo.2005.11.013 (2006).

[b17] Peterson R. N. *et al.* Determination of transport rates in the Yellow River-Bohai Sea mixing zone via natural geochemical tracers. Cont. Shelf. Res. 28, 2700–2707, 10.1016/j.csr.2008.09.002 (2008).

[b18] Peterson R. N. *et al.* Radon and radium isotope assessment of submarine groundwater discharge in the Yellow River delta, China. J. Geophys. Res. 113, C09021, 10.1029/2008JC004776 (2008).

[b19] Taniguchi M. *et al.* Submarine groundwater discharge from the Yellow River Delta to the Bohai Sea, China. J. Geophys. Res. 113, C06025, 10.1029/2007JC004498(2008).

[b20] Xu B. *et al.* Hydrodynamics in the Yellow River Estuary via radium isotopes: Ecological perspectives. Cont. Shelf. Res. 66, 19–28, 10.1016/j.csr.2013.06.018 (2013).

[b21] Xu B. *et al.* Natural 222Rn and 220Rn indicate the impact of the Water–Sediment Regulation Scheme (WSRS) on submarine groundwater discharge in the Yellow River estuary, China. Appl. Geochem. 51, 79–85, 10.1016/j.apgeochem.2014.09.018 (2014).

[b22] Moore W. S. Large groundwater inputs to coastal waters revealed by Ra-226 enrichments. Nature 380, 612–614, 10.1038/380612a0 (1996).

[b23] Monsen N. E., Cloern J. E., Lucas L. V. & Monismith S. G. A comment on the use of flushing time, residence time, and age as transport time scales. Limnol. Oceanogr. 47, 1545–1553 (2002).

[b24] Moore W. S., Blanton J. O. &Joye S. B. Estimates of flushing times, submarine groundwater discharge, and nutrient fluxes to Okatee Estuary, South Carolina. J. Geophys. Res. 111, C09006, 10.1029/2005JC003041 (2006).

[b25] Sanford L. P., Boicourt W. C. & Rives S. R. Model for estimating tidal flushing of small embayments. J. Waterw. Port C.-ASCE 118, 635–654 (1992).

[b26] Hainbucher D., Hao W., Pohlmann T., Sundermann J. & Feng S. Z. Variability of the Bohai Sea circulation based on model calculations. J. Marine Syst. 44, 153–174, 10.1016/j.jmarseys.2003.09.008 (2004).

[b27] Wei H., Hainbucher D., Pohlmann T., Feng S. Z. &Suendermann J. Tidal-induced Lagrangian and Eulerian mean circulation in the Bohai Sea. J. Marine Syst. 44, 141–151, 10.1016/j.jmarseys.2003.09.077 (2004).

[b28] Wang Q., Guo X. & Takeoka H. Seasonal variations of the Yellow River plume in the Bohai Sea: A model study. J. Geophys. Res. 113, C08046, 10.1029/2007JC004555 (2008).

[b29] Li H. & Jiao J. Quantifying tidal contribution to submarine groundwater discharges: A review. Chin. Sci. Bull. 58, 3053–3059, 10.1007/s11434-013-5951-7 (2013).

[b30] Xin P., Robinson C., Li L., Barry D. A. & Bakhtyar R. Effects of wave forcing on a subterranean estuary. Water Resour. Res. 46 10.1029/2010wr009632 (2010).

[b31] Xin P. *et al.* Memory of past random wave conditions in submarine groundwater discharge. Geophys. Res. Lett. 41, 2401–2410, 10.1002/2014gl059617 (2014).

[b32] Smith A. J. Mixed convection and density-dependent seawater circulation in coastal aquifers. Water Resour. Res. 40 10.1029/2003wr002977 (2004).

[b33] Robinson C., Gibbes B., Carey H. & Li L. Salt-freshwater dynamics in a subterranean estuary over a spring-neap tidal cycle. J. Geophys. Res. 112, C09007, 09010.01029/02006JC003888. (2007).

[b34] Qu W., Li H., Wan L., Wang X. & Jiang X. Numerical simulations of steady-state salinity distribution and submarine groundwater discharges in homogeneous anisotropic coastal aquifers. Adv. Water Res. 74, 318–328, 10.1016/j.advwatres.2014.10.009 (2014).

[b35] Wilson A. M. The occurrence and chemical implications of geothermal convection of seawater in continental shelves. Geophys. Res. Lett. 30 10.1029/2003gl018499 (2003).

[b36] Konikow L. F., Akhavan M., Langevin C. D., Michael H. A. & Sawyer A. H. Seawater circulation in sediments driven by interactions between seabed topography and fluid density. Water Resour. Res. 49, 1386–1399, 10.1002/wrcr.20121 (2013).

[b37] Wilson A. M. Fresh and saline groundwater discharge to the ocean: A regional perspective. Water Resour. Res. 41, W02016, 10.1029/2004wr003399 (2005).

[b38] Han D. M., Song X. F., Currell M. J. & Tsujimura M. Using chlorofluorocarbons (CFCs) and tritium to improve conceptual model of groundwater flow in the South Coast Aquifers of Laizhou Bay, China. Hydrol. Process. 26, 3614–3629, 10.1002/hyp.8450 (2012).

[b39] Tang Q. S. & Meng T. X. Atlas of the ecological environment and living resources in the Bohai Sea. (Qingdao Publishing House, Qingdao, China1997) (in Chinese).

[b40] Swarzenski P. W., Reich C., Kroeger K. D. & Baskaran M. Ra and Rn isotopes as natural tracers of submarine groundwater discharge in Tampa Bay, Florida. Mar. Chem. 104, 69–84, 10.1016/j.marchem.2006.08.001 (2007).

[b41] Hwang D. W., Kim G., Lee W. C. & Oh H. T. The role of submarine groundwater discharge (SGD) in nutrient budgets of Gamak Bay, a shellfish farming bay, in Korea. J. Sea Res. 64, 224–230 (2010).

[b42] Kazemi G. A. Editor's Message: Submarine groundwater discharge studies and the absence of hydrogeologists. Hydrogeol. J. 16, 201–204, 10.1007/s10040-007-0251-4 (2008).

[b43] Moore W. S. Seasonal distribution and flux of radium isotopes on the southeastern U.S. continental shelf. J. Geophys. Res. 112 10.1029/2007jc004199 (2007).

[b44] Moore W. S. Sampling 228Ra in the deep ocean. Deep Sea Research and Oceanographic Abstracts 23, 647–651, 10.1016/0011-7471(76)90007-3 (1976).

[b45] Kim G., Burnett W. C., Dulaiova H., Swarzenski P. W. & Moore W. S. Measurement of Ra-224 and Ra-226 activities in natural waters using a radon-in-air monitor. Environ. Sci. Technol. 35, 4680–4683, 10.1021/es010804u (2001). 11770771

[b46] Lee C. M., Jiao J. J., Luo X. & Moore W. S. Estimation of submarine groundwater discharge and associated nutrient fluxes in ToloHarbour, Hong Kong. Sci. Total Environ. 433, 427–433, 10.1016/j.scitotenv.2012.06.073 (2012). 22819893

[b47] Moore W. S. & Arnold R. Measurement of Ra-223 and Ra-224 in coastal waters using a delayed coincidence counter. J. Geophys. Res. 101, 1321–1329, 10.1029/95jc03139 (1996).

