# Supplementary material for: Submarine fresh groundwater discharge into Laizhou Bay comparable to the Yellow River flux
Source: Sci Rep. 2015 Mar 6;5:8814. doi: 10.1038/srep08814 (PMC4649716; doi:10.1038/srep08814)
Supplement: Supplementary Information [file srep08814-s1.doc]

**Supplementary Information**

**Submarine fresh groundwater discharge into Laizhou Bay comparable to the Yellow River flux**

Xuejing Wang ^1,2^, Hailong Li ^1,2^, [[1]](#footnote-2), Jiu Jimmy Jiao ^3^, D. A. Barry^4^, Ling Li^5, 6^,

Xin Luo ^3^,Chaoyue Wang ^2^, Li Wan ^2^, Xusheng Wang ^2^ , Xiaowei Jiang ^2^, Qian Ma ^2^,

Wenjing Qu ^2^

^1^ State Key Laboratory of Biogeology and Environmental Geology, China University of Geosciences, Beijing 100083, China

^2^ MOE Key Laboratory of Groundwater Circulation & Environment Evolution and School of Water Resources and Environment, China University of Geosciences, Beijing 100083, China

^3^ Department of Earth Sciences, The University of Hong Kong, Hong Kong, China

^4^ Laboratoire de technologie écologique, Institut d’ingénierie de l’environnement, Faculté de l’environnement naturel, architectural et construit, Ecole Polytechnique Fédérale de Lausanne, Lausanne, 1015 Lausanne, Switzerland

^5^ School of Civil Engineering, University of Queensland, Brisbane, Queensland

^6^ State Key Laboratory of Hydrology-Water Resources and Hydraulic Engineering, Hohai University, Nanjing, China

**Flushing Time Model**

For a bay system under steady state, one can ignore the storage variation so that the additions of radium (Ra) are balanced by losses. Neglecting the effects of wind, waves, and storms, the main processes causing Ra loss in the system are tidal effect and decay in the case of short-lived Ra. The ^226^Ra balance can be written as:

$F{}^{226}Ra=\frac{P_{\text{tide}}(1-b)I{}^{226}Ra}{T_{\text{tide}}V_{Bay}}$ (S1)

where *F*^226^Ra is the total flux of ^226^Ra into the bay, *I*^226^Ra is the inventory of ^226^Ra in the bay, $P_{\text{tide}}$ is the tidal prism (total volume of seawater entering the bay during a rising tide), *b* is return flow factor (percentage of the tidal prism that returns from outside of the bay during a rising tide), $T_{\text{tide}}$ is the tidal period, *V_Bay_* is the volume of water in the Bay. Here we followed Sanford et al.^1^ to quantify the renewal rate $Q$ of the seawater in the bay using:

$Q=\frac{P_{\text{tide}}(1-b)}{T_{\text{tide}}}$ (S2)

Concerning the change of ^226^Ra inventory for a bay during a tidal cycle, the returning portion of seawater $P_{\text{tide}}b$ makes no changes to the ^226^Ra inventory, while the renewed portion causes a loss of $P_{\text{tide}}(1-b)I{}^{226}Ra/V_{Bay}$. This leads to equation (S1).

Considering the decay, we can write a similar equation for ^223^Ra:

$F{}^{223}Ra=\left[\frac{P_{\text{tide}}(1-b)}{T_{\text{tide}}V_{Bay}}+\lambda_{223}\right]I{}^{223}Ra$ (S3)

where $\lambda_{223}$is the decay constant for ^223^Ra and equals 0.061 d^-1^.

Dividing equation (S3) by equation (S1) yields:

$F({}^{223}Ra/{}^{226}Ra)=\left[\frac{T_{\text{tide}}V_{Bay}}{P_{\text{tide}}(1-b)}\lambda_{223}+1\right]I({}^{223}Ra/{}^{226}Ra)$ (S4)

where *F*(^223^Ra/^226^Ra) is the ^223^Ra/^226^Ra activity ratio of the flux into the bay, *I*(^223^Ra/^226^Ra) is the ^223^Ra/^226^Ra activity ratio in the bay.

The term $T_{\text{tide}}V_{Bay}/[P_{\text{tide}}(1-b)]$ in equation (S4) is the flushing time $T_{f}$^1^. Thus we can rearrange equation (S4) and solve it for $T_{f}$:

$T_{f}=\frac{T_{\text{tide}}V_{Bay}}{P_{\text{tide}}(1-b)}=\frac{1}{\lambda_{223}}\left[\frac{F({}^{223}Ra/{}^{226}Ra)}{I({}^{223}Ra/{}^{226}Ra)}-1\right]$ (S5)

Equation (S5) is reproduced as equation (1) in the main text. Note that equation (S5) is a combination of the flushing time given by equation (2) in Moore et al.^2^ and the apparent water age given by equation (14) in Moore et al.^2^. From above assumptions and derivation, one can consider the apparent water age as the flushing time.

In equation (S5) or (1), the ^223^Ra/^226^Ra activity ratio can also be replaced with any of the ^223^Ra/^228^Ra, ^224^Ra/^228^Ra, and ^224^Ra/^226^Ra activity ratios to calculate the flushing time. Here we used the ^223^Ra/^226^Ra activity ratio because the scale of the flushing time for Laizhou Bay is appropriately comparable to the ^223^Ra half-life of 11.4 d. Figure S1 shows plots of ^223^Ra versus ^226^Ra in seawater samples. We collected eight groundwater samples (see G1 to G8 in Table S1). The activities of ^223^Ra and ^226^Ra of samples G1 and G2 are abnormally higher than those of the other samples. The reason for this is most probably that the samples G1 and G2 were collected from very shallow groundwater in upper intertidal zone where the groundwater composition was affected by strong evaporation. Thus, we excluded the samples G1 and G2 and used the other 6 groundwater samples (Table S1) to estimate the ^223^Ra/^226^Ra activity ratio *F*(^223^Ra/^226^Ra) of the flux into the Bay. The result is:

*F*(^223^Ra/^226^Ra)$=\frac{\text{Averaged }^{223}\text{Ra of groundwater samples at stations G3\textasciitilde G8}}{\text{Averaged }^{226}\text{Ra of groundwater samples at stations G8\textasciitilde G8}}$=0.084

The inventory ^223^Ra/^226^Ra activity ratio in the Bay is:

*I*(^223^Ra/^226^Ra)=$\frac{\text{Inventory of }^{223}\text{Ra in the Bay}}{\text{Inventory of }^{226}\text{Ra in the Bay}}$= 0.027.

Here the “Inventory of ^223^Ra in the Bay” was calculated as the sum of the ^223^Ra inventory in the seawater prism corresponding to the triangular elements in Fig. S2. The ^223^Ra (or ^226^Ra) inventory in each seawater prism was calculated as the product of the volume of the prism and the average of the observed ^223^Ra (or ^226^Ra) activities at three vertexes of the triangle element. The volume of each seawater prism was calculated as the product of the area of the corresponding triangle element and the averaged seawater depths at the three vertexes of the triangle element.


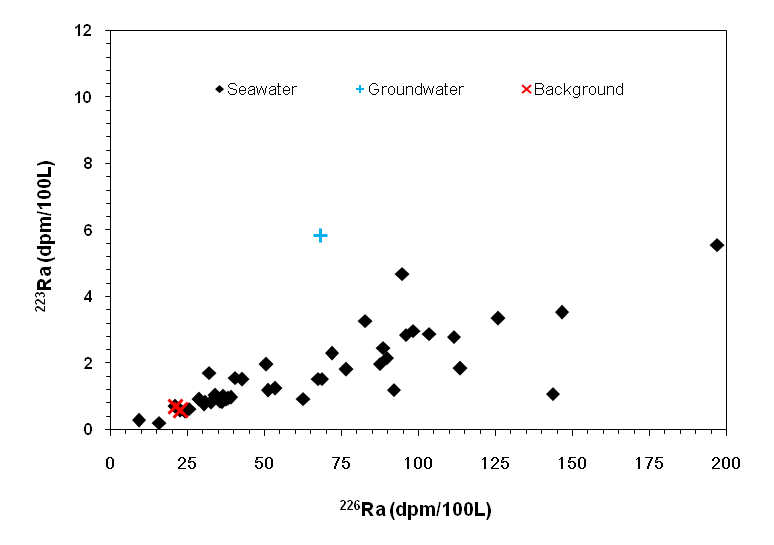


**Figure S1. ^223^Ra versus ^226^Ra for seawater samples**. Values at the stations S3 and S4 are marked with the red crosses to represent approximately the background values of the seawater in the open sea. Averaged values of ^223^Ra and ^226^Ra (blue cross) for the six groundwater samples at G3 ~ G8 are also plotted for comparison.


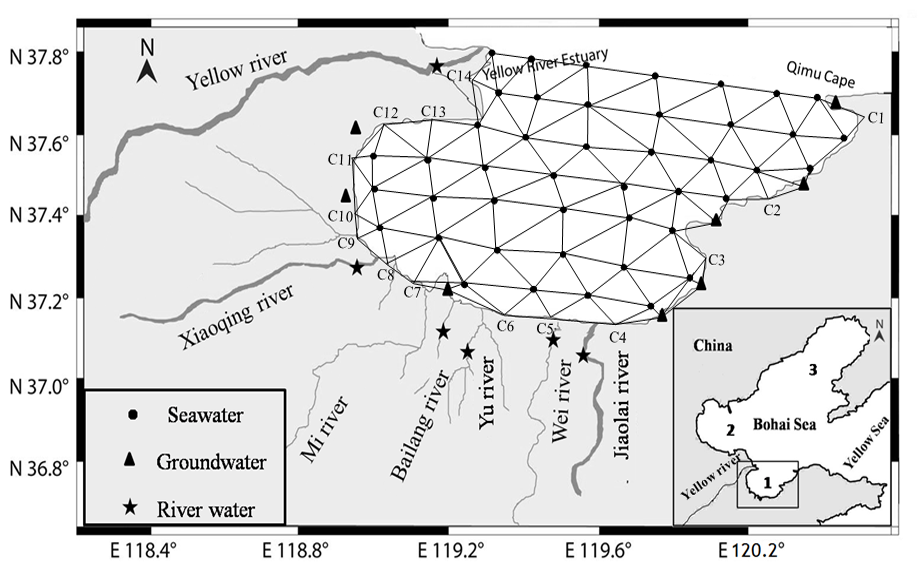


**Figure S2. Triangle elements for calculations of the area of Laizhou Bay, the water volume, salt mass, and inventories of ^223^Ra and ^226^Ra**. Seawater depths at triangle nodes on the coastline were zero. At each triangle node on the coastline (number C1-C14) where no measurements were conducted for salinity and Ra activities, the values of salinity and Ra activities were approximated by those at the nearest seawater observation station. Details of all the triangle nodes are given in Table S1, which include the location coordinates, seawater depths, salinity, and activities of ^223^Ra and ^226^Ra. Maps were created with MAPGIS 6.7 software.

Substituting the above values of *I*(^223^Ra/^226^Ra) and *F*(^223^Ra/^226^Ra) into equation (S5) gives the flushing time of the coastal water as $T_{f}$=36.6 d. Equation (S5) indicates that the flushing time $T_{f}$ of the coastal water is sensitive to the value of *F*(^223^Ra/^226^Ra). This uncertainty was considered following the approach of Moore et al.^2^ with a variation in *F*(^223^Ra/^226^Ra) by ± 10% of its value of 0.084, which resulted in a change in the flushing time of ± 5.3 d. So our ^223^Ra/^226^Ra estimate of the flushing time is $T_{f}$=36.6 ± 5.3 d.

Hainbucher et al.^3^, who modeled seawater circulation in the Bohai Sea, determined that its flushing time is 0.5 - 1 y. Laizhou Bay is about one-eleventh of the Bohai Sea by surface area, so one approach is to approximate the flushing time of the Bay proportionally, i.e., 18-36 d. Another approach is to use Figure 10 of Hainbucher et al.^3^, which shows the flushing time (defined as “turnover time” in their paper) in the different area of the Bohai Sea. This figure suggests the Laizhou Bay’s flushing time in the range of 15-40 d. Peterson et al.^4^ and Xu et al.^5^ used an “apparent Ra age model” to estimate the transport of surface seawater in the Yellow River estuary and derived the transport rate ranging from 3 to 7 km/d. Using this water transport rate and an average transport path length of 120 km for Laizhou Bay, we can obtain an approximate estimation of the flushing time ranging from 17 to 40 d. Here Laizhou Bay is approximately regarded as a semi-circle, so the average transport path length may be approximated by the diameter of the semi-circle (120 km). Our estimate of *T_f_* based on equation (S5) is consistent with these studies.

Based on equation (S5) and the flushing time ($T_{f}$) derived from the Ra method, we can estimate the return flow factor *b*, which is given by $b=1-T_{\text{tide}}V_{Bay}/P_{\text{tide}}T_{f}$. The tidal prism ($P_{\text{tide}}$) can be approximated by a value of 5.40 × 10^9^ m^3^, the product of the average tidal range (0.9 m) and the area of the Bay (~6000 km^2^). The total volume of water in the Bay $V_{Bay}=$4.72 × 10^10^ m^3^ was calculated as the sum of the volumes of all the seawater prisms corresponding to the triangle elements in Fig. S2. The tidal period $T_{\text{tide}}$ is 0.5 d. Using these values yields a return flow factor of *b* = 0.88. This indicates that major portion of the tidal prism returns from outside of the Bay during the rising tide.

**Water and Salt Mass Balance Model**


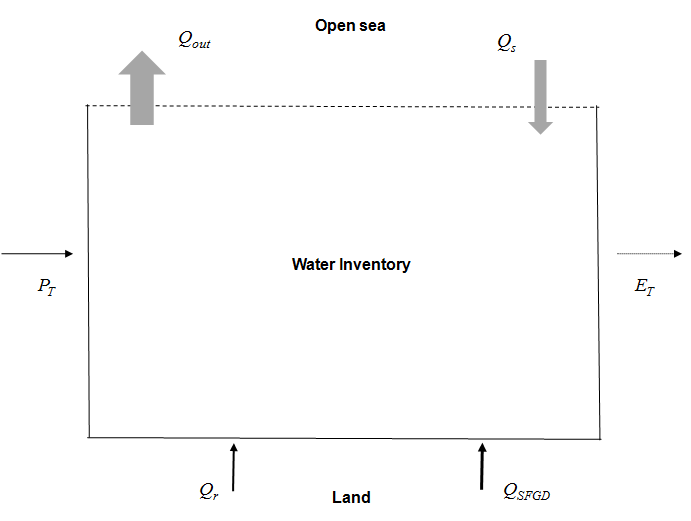


**Figure S3. Schematic of the water mass balance model of the Bay.** Water volume is a function of river discharge ($Q_{r}$), SFGD ($Q_{SFGD}$), precipitation ($P_{T}$), inflows of outside seawater ($Q_{s}$), evaporation ($E_{T}$), and outflows to the open sea ($Q_{out}$).

Figure S3 shows a conceptual water mass balance model for the Bay. The inputs of water should be from seawater outside of the Bay ($Q_{s}$), precipitation ($P_{T}$), and freshwater discharge ($Q_{f}$) including the river flux ($Q_{r}$) and submarine fresh groundwater discharge ($Q_{SFGD}$). The outputs of water include outflow to the open sea ($Q_{out}$) and evaporation ($E_{T}$). Neglecting the net variation of water storage with time (i.e., over a tidal period) in this system, the total water inflow is equal to the total outflow. Thus the water balance can be represented by the following equation:

$Q_{s}+Q_{f}+P_{T}=Q_{out}+E_{T} ,$ (S6a)

where

$Q_{f}=Q_{r}+Q_{SFGD},$ (S6b)

$Q_{out}=V_{Bay}/T_{f}$ (S6c)

The inventory of salt in the Bay is a result of mixing between a volume of freshwater and a volume of seawater in the open sea (i.e., representative seawater from outside the Bay). It can be represented by:

$M_{s}=s_{f}V_{f}+s_{s}V_{s}$ (S7a)

where $M_{s}$ is the total salt mass stored in the Bay;$V_{f}$ and $V_{s}$ are the volumes of freshwater and the open-sea seawater in the Bay, respectively; $s_{f}$ and $s_{s}$ are the salinity of freshwater and the open-sea seawater, respectively. Since the salinity of the freshwater $s_{f}$ is zero, so from equation (S7a) one has

$V_{s}=M_{s}/s_{s}$ (S7b)

On the other hand, from the definition of the flushing time, we have $Q_{s}=V_{s}/T_{f}$. Using equation (S7b), one has

$Q_{s}=\frac{M_{s}}{T_{f}s_{s}}$ (S7c)

Substituting equations (S6b), (S6c), and (S7c) back into equation (S6a), and solving for $Q_{FSGD}$ yields

$Q_{SFGD}=\frac{1}{T_{f}}\left[V_{Bay}-\frac{M_{s}}{s_{s}}\right]+(E_{T}-P_{T})-Q_{r}$ (S8)

Finally, since the river flux $Q_{r}$ is defined as

$Q_{r}=\sum_{\text{Other} \text{rivers}} Q_{r,i}+r_{YL}Q_{Y}$, (S9)

substitution of equation (S9) into equation (S8) gives equation (2).

The value of $M_{s}$=1.22×10^12^ m^3^psu was calculated as the sum of the salt mass of all the seawater prisms corresponding to the triangle elements in Fig. S2. The salt mass of each seawater prism was calculated as the product of the volume of the prism and the average of the observed seawater salinities at three vertexes of the triangle element. Note that the unit of the salt mass $M_{s}$ is m^3^psu since the unit of salinity that we use here is psu. The salinity value of open-sea seawater salinity $s_{s}$=27.34 psu was approximated by the average of observed salinities at the two stations S3 and S4, which are closest to the open sea and farthest from the coastline among all the offshore stations (Fig. 1).

The evaporation and precipitation parameter $E_{T}-P_{T}$=$A_{LZ}(E-P)$, where $A_{LZ}=$5999.57 km^2^ is the area of Laizhou Bay, which was calculated as the sum of the area of each of the triangle elements in Fig. S2, $E$ and $P$ are the sum of the daily evaporation and precipitation (Fig. S4) occurred during the flushing time ($T_{f}$ d) immediately before the observation time of 22 August 2012.

The value of the Yellow River flux, $Q_{Y}$, was calculated as the weighted average of the monthly discharge in July and August 2012 corresponding to the flushing time ($T_{f}$ d immediately before the observation time). The weight for each month was set to be the number of days of the month occupied by the flushing time. Because the annual average flux of the Yellow River in 2012 was more than 20 times of the sum of fluxes of all the other rivers flowing into the Bay, we only used the annual average flux for other rivers (Table S2). The values of all the parameters used in equation (2) are summarized in Table S3.


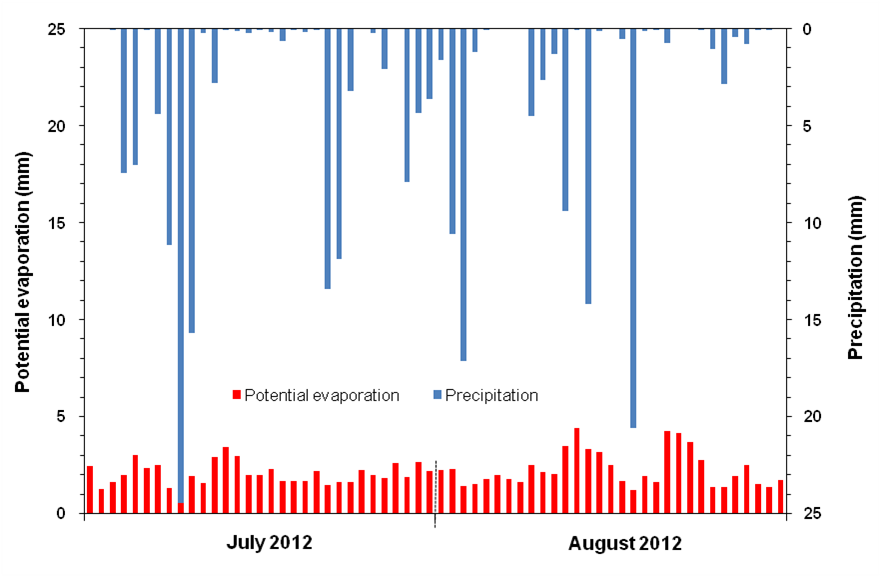


**Figure S4. Daily precipitation and potential evaporation of Laizhou Bay in July and August 2012**. Data from the European Centre for Medium-Range Weather Forecasts.

**^226^Ra Mass Balance Model**

Figure S5 shows a general conceptual mass balance model for Ra in the Bay. The inputs of Ra are from discharge waters (rivers and SGD), desorption from suspended particles and diffusion from bottom sediments. The losses of Ra include mixing with open sea and radioactive decay. The fluxes of Ra supplied by precipitation and atmospheric dusts do not significantly affect the result and are neglected^6^. Using the steady state premise, one can ignore the variation of ^226^Ra storage in the Bay. Thus the gain of Ra is equal to the loss^3^. The general mass balance equation for Ra can be described by the following expression:

$Q_{SGD}Ra_{gw}+\sum(Ra_{r,i}Q_{r,i}+M_{sus,i}\alpha_{des,i})+S_{sub}\alpha_{diff}=\frac{V_{Bay}\text{(}Ra_{Bay}-Ra_{op}\text{)}}{T_{f}}+\lambda V_{Bay}Ra_{Bay}$ (S10a)

where $Ra_{gw}$, $Ra_{r,i}$, $Ra_{Bay}$, and $Ra_{op}$ are the Ra activity [dpmL^-3^] in groundwater, water from the *i*^th^ river, Bay water and seawater outside of the Bay (referred to as “open sea water” hereafter for convenience), respectively; $Q_{SGD}$, and $Q_{r,i}$ are the volumetric fluxes of SGD and runoff of the *i*^th^ river into the Bay, respectively, [L^3^T^-1^]; $M_{sus,i}$ is the mass of the suspended particles in the *i*^th^ river, [MT^-1^]; $\alpha_{des,i}$ is the constant desorption parameter [dpmM^-1^] of the *i*^th^ river; $\alpha_{diff}$ is the diffusion coefficient [dpmL^-2^T^-1^]; $S_{sub}$ is the bottom area of the Bay, [L^2^]; and $\lambda$ is the decay constant of the considered Ra isotope, [T^-1^].


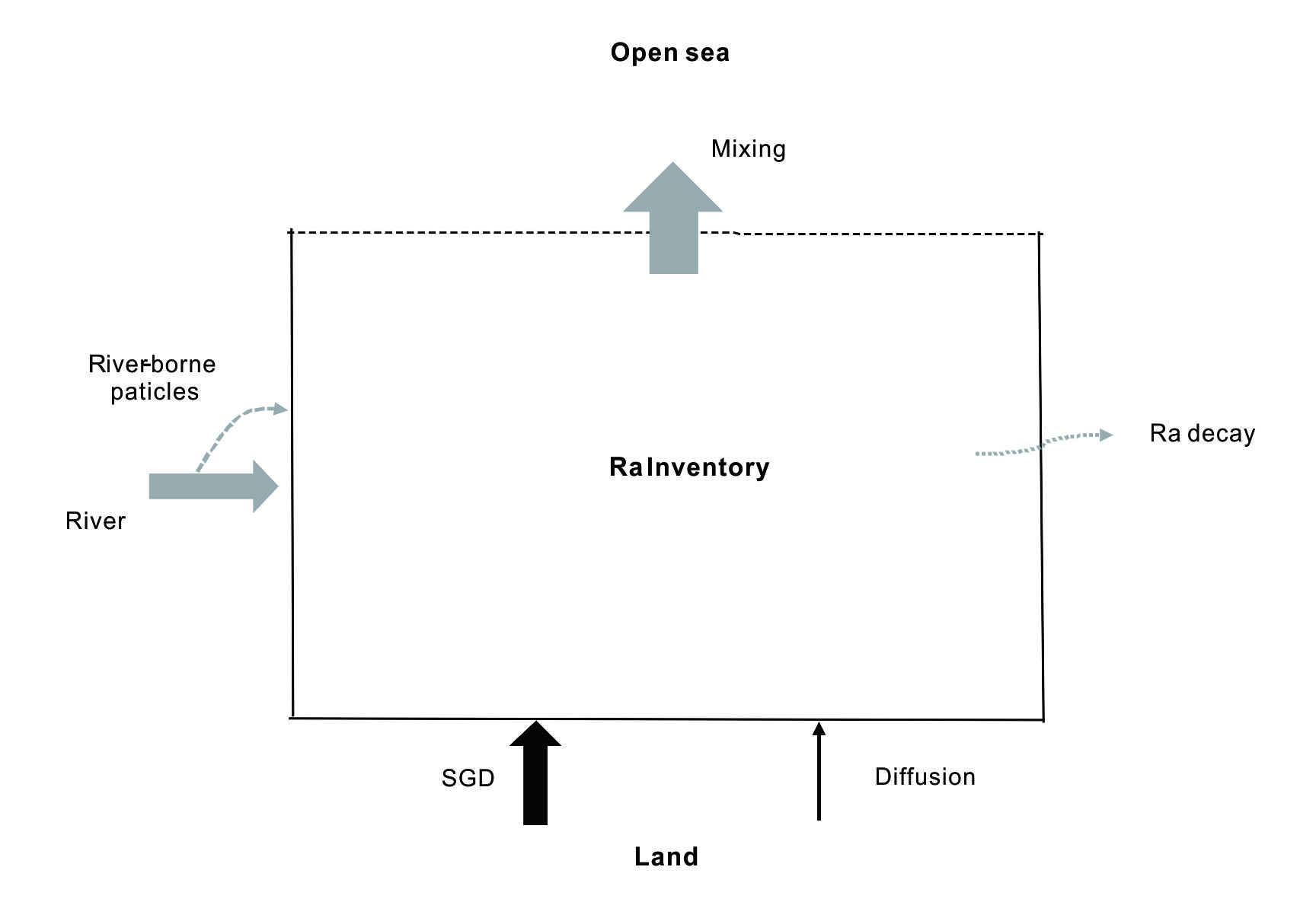


**Figure S5. A general conceptual mass balance model of Ra isotopes.** The fluxes of Ra supplied by precipitation and atmospheric dusts are neglected.

Here we used the long-lived ^226^Ra to establish the mass balance model for Laizhou Bay. Due to the long half-life ($\lambda_{226}=1.186\times10^{\text{-}6}$ d^-1^), we neglected the decay of ^226^Ra (0.1‰ of the total losses) and considered mixing as the only sink. Thus, based on equation (S10a) the mass balance equation for ^226^Ra in the Bay is:

$Q_{SGD}^{226}Ra_{gw}+r_{YL} ^{226} Ra_{TYL}+^{226} Ra_{Tr}=\frac{V_{Bay}}{T_{f}}(^{226}Ra_{Bay}-^{226} Ra_{bg})$ (S10b)

where

$^{226} Ra_{TYL}$ $= ^{226} Ra_{YL}Q_{Y}+M_{sus,YL}\alpha_{des,YL}$ (S10c)

is the total input of ^226^Ra from the Yellow River discharge and suspended particle desorption, with ${}^{226}Ra_{YL}$ being the ^226^Ra activity of the Yellow River water, $M_{sus,YL}$ and $\alpha_{des,YL}$ being the mass flux [MT^-1^] of the suspended particles transported by the Yellow River and the desorption parameter [dpmM^-1^], respectively; and

$^{226} Ra_{Tr}=\sum_{\text{Other} \text{rivers}} (^{226}Ra_{r,i}Q_{r,i}+M_{sus,i}\alpha_{des,i})$ (S10d)

is the total input of ^226^Ra from discharges and suspended particle desorption of all rivers other than the Yellow River. The term ${}^{226}Ra_{bg}$ represents the background activity of ^226^Ra. Because of the complexes of the mixing between the Bay water and the open sea water, the “end-member” value of ^226^Ra activity in open sea water ($Ra_{op}$) is difficult to determine in this case. Following the approach of Peterson et al.^7^, we use the average of two low-activity values of ^226^Ra measured at stations S3 and S4 (Fig. 1) as an indicator of the background activity. This background activity, however, already contains the contribution from bottom sediments, so we neglected the Ra diffusion term $S_{sub}\alpha_{diff}$ from the bottom sediments in equation (S10b). From equation (S10b), we can solve for the SGD flux and obtain equation (3).

The discharges of the major rivers entering the Bay and the concentrations of river-borne particles during the study periods are listed in Table S2. All parameters needed in the model and the calculations of ^226^Ra flux are listed in Table S4. The ^226^Ra background value of 21.89 dpm/100L was calculated as the average of the two low-activity samples at stations S3 and S4. The coefficient of desorption of ^226^Ra ($\alpha_{des,i}$) from the river-borne particles was set to be 600 dpm/kg for all the rivers^7^.

**References**

1. S anford, L.P., Boicourt, W.C. & Rives, S.R. Model for estimating tidal flushing of small embayments. *J. Waterw. Port C.-ASCE* **118**, 635-654 (1992).
2. Moore, W.S., Blanton, J.O. &Joye, S.B. Estimates of flushing times, submarine groundwater discharge, and nutrient fluxes to Okatee Estuary, South Carolina. *J. Geophys. Res.* **111**,C09006, doi:10.1029/2005JC003041 (2006).
3. Hainbucher, D., Hao, W., Pohlmann, T., Sundermann, J. & Feng, S.Z. Variability of the Bohai Sea circulation based on model calculations. *J. Marine Syst.* **44**, 153-174, doi:10.1016/j.jmarseys.2003.09.008 (2004).
4. Peterson, R.N. *et al.* Determination of transport rates in the Yellow River-Bohai Sea mixing zone via natural geochemical tracers. *Cont. Shelf. Res.* **28**, 2700-2707, doi:10.1016/j.csr.2008.09.002 (2008a).
5. Xu, B. *et al.* Hydrodynamics in the Yellow River Estuary via radium isotopes: Ecological perspectives. *Cont. Shelf. Res.* **66**, 19-28, doi:10.1016/j.csr.2013.06.018 (2013).
6. Moore, W.S., Sarmiento, J.L. & Key, R.M. Submarine groundwater discharge revealed by 228Ra distribution in the upper Atlantic Ocean. *Nat. Geosci.* **1**, 309-311, doi:10.1038/ngeo183 (2008).
7. Peterson, R.N. *et al.* Radon and radium isotope assessment of submarine groundwater discharge in the Yellow River delta, China. *J. Geophys. Res.* **113**, C09021,doi:10.1029/2008JC004776 (2008b).

**Table S1** All samples collected for measurements of Ra isotopes.

| Stations | Longitude | Latitude | Water depth | Salinity | Salinity | ^223^Ra | ^226^Ra |
| --- | --- | --- | --- | --- | --- | --- | --- |
|  |  |  | (m) | (2012) | (2014) | (dpm/100L) | |
| S1 | 120.20° | 37.69° | 22.0 | 27.60 | 30.02 | 0.84±0.10 | 35.67±2.93 |
| S10 | 119.59° | 37.66° | 13.6 | 26.61 | 28.76 | 0.81±0.10 | 30.55±2.93 |
| S11 | 119.77° | 37.65° | 13.2 | 27.27 | 28.94 | 1.52±0.18 | 68.54±3.58 |
| S12 | 119.97° | 37.63° | 13.3 | 25.96 | 29.57 | 0.27±0.03 | 9.15±2.93 |
| S13 | 120.13° | 37.61° | 12.0 | 27.42 | 29.49 | 0.90±0.11 | 28.77±2.93 |
| S14 | 120.27° | 37.59° | 8.7 | 26.78 | 29.47 | 1.55±0.19 | 40.45±2.60 |
| S15 | 120.17° | 37.51° | 1.0 | 27.62 | 29.60 | 0.92±0.11 | 62.59±3.90 |
| S16 | 120.03° | 37.51° | 12.2 | 27.27 | 29.18 | 0.82±0.10 | 32.67±3.58 |
| S17 | 119.92° | 37.54° | 11.9 | 27.17 | 29.97 | 0.19±0.02 | 15.77±1.69 |
| S18 | 119.75° | 37.56° | 13.6 | 27.14 | 28.70 | 0.94±0.11 | 34.77±3.58 |
| S19 | 119.58° | 37.57° | 13.2 | 26.54 | 28.31 | 1.18±0.14 | 51.10±5.20 |
| S2 | 120.09° | 37.70° | 14.5 | 27.00 | 30.03 | 0.75±0.09 | 30.25±3.59 |
| S20 | 119.41° | 37.60° | 13 | 24.29 | 27.58 | 1.80±0.22 | 76.52±5.53 |
| S21 | 119.28° | 37.61° | 4.5 | 24.34 | 27.78 | 2.29±0.27 | 71.82±5.53 |
| S22 | 119.01° | 37.54° | 3.0 | 24.21 | 27.34 | 2.88±0.35 | 103.37± 6.18 |
| S23 | 119.16° | 37.53° | 5.0 | 24.47 | 27.65 | 2.79±0.33 | 111.32±6.83 |
| S24 | 119.30° | 37.51° | 8.0 | 24.25 | 27.70 | 5.56±0.67 | 196.78±8.13 |
| S25 | 119.49° | 37.49° | 11.1 | 26.01 | 26.75 | 0.54±0.06 | 24.27±5.20 |
| S26 | 119.67° | 37.47° | 13.8 | 26.37 | 27.63 | 0.61±0.07 | 25.48±4.88 |
| S27 | 119.81° | 37.45° | 9.3 | 26.82 | 28.22 | 1.02±0.12 | 33.94±2.93 |
| S28 | 119.95° | 37.44° | 10.0 | 27.72 | 27.63 | 1.00±0.12 | 36.69±6.18 |
| S29 | 119.81° | 37.36° | 3.0 | 27.35 | 27.07 | 1.19±0.14 | 91.89±2.93 |
| S3^a^ | 119.94° | 37.72° | 15 | 27.24 | 30.02 | 0.69±0.08 | 21.07±3.79 |
| S30 | 119.68° | 37.38° | 8.3 | 25.8 | 27.07 | 1.70±0.20 | 31.99±3.25 |
| S31 | 119.50° | 37.40° | 11.0 | 25.84 | 27.47 | 0.82±0.10 | 36.15±4.23 |
| S32 | 119.32° | 37.42° | 10.2 | 24.64 | 27.37 | 1.23±0.15 | 53.40±5.85 |
| S33 | 119.17° | 37.43° | 3.8 | 23.59 | 27.32 | 0.97±0.12 | 39.03±6.18 |
| S34 | 119.00° | 37.45° | 2.0 | 23.34 | 27.74 | 3.34±0.4 | 125.77±6.18 |
| S35 | 119.02° | 37.35° | 3.5 | 21.48 | 27.30 | 4.68±0.56 | 94.66±3.58 |
| S36 | 119.18° | 37.34° | 2.0 | 22.19 | 27.08 | 2.16±0.26 | 89.69±5.20 |
| S37 | 119.33° | 37.32° | 5.2 | 23.86 | 27.14 | 1.95±0.23 | 50.54±4.88 |
| S38 | 119.51° | 37.30° | 8.5 | 24.33 | 27.40 | 1.07±0.13 | 143.45±1.14 |
| S39 | 119.67° | 37.28° | 6.1 | 25.33 | 27.38 | 1.50±0.18 | 42.81±3.25 |
| S4^a^ | 119.77° | 37.74° | 15.3 | 27.43 | 29.31 | 0.56±0.07 | 22.70±4.01 |
| S40 | 119.83° | 37.26° | 4.5 | 26.97 | 27.05 | 1.97±0.24 | 87.34±3.25 |
| S41 | 119.72° | 37.18° | 2.1 | 26.23 | 27.57 | 3.52±0.42 | 146.43±4.88 |
| S42 | 119.57° | 37.20° | 3.4 | 23.10 | 28.57 | 0.91±0.11 | 37.48±4.55 |
| S43 | 119.42° | 37.22° | 6.2 | 23.80 | 27.45 | 3.27±0.39 | 82.59±3.58 |
| S44 | 119.24° | 37.23° | 5.4 | 22.15 | 27.88 | 2.46±0.29 | 88.54±3.25 |
| S5 | 119.59° | 37.76° | 15.0 | 26.34 | 28.61 | 0.93±0.11 | 38.22±4.44 |
| S6 | 119.44° | 37.78° | 15.0 | 25.46 | 28.22 | 1.51±0.18 | 58.69±5.12 |
| S7 | 119.33° | 37.79° | 4.0 | 24.98 | 27.00 | 2.84±0.34 | 95.84±5.20 |
| S8 | 119.33° | 37.69° | 5.0 | 23.07 | 27.24 | 2.96±0.36 | 98.15±7.15 |
| S9 | 119.45° | 37.68° | 13.0 | 23.89 | 27.37 | 1.84±0.22 | 113.50±5.53 |
| G1 | 120.25° | 37.67° | na | 30.98 | 33.16 | 45.20±3.16 | 190.13±11.70 |
| G2 | 118.92° | 37.44° | na | 33.10 | 30.24 | 22.40±1.57 | 118.49±9.33 |
| G3^b^ | 118.96° | 37.62° | na | 33.10 | na | 10.76±0.75 | 54.42±5.32 |
| G4^b^ | 120.15° | 37.47° | na | 27.42 | 31.56 | 1.14±0.08 | 53.72± 5.16 |
| G5^b^ | 119.92° | 37.38° | na | 22.35 | 29.06 | 1.77±0.12 | 43.96±5.51 |
| G6^b^ | 119.87° | 37.23° | na | 25.33 | 28.61 | 11.29±0.79 | 97.04±6.47 |
| G7^b^ | 119.78° | 37.16° | na | 17.08 | 32.54 | 4.50±0.31 | 84.92±9.35 |
| G8^b^ | 119.21° | 37.22° | na | 22.35 | 31.94 | 5.57±0.39 | 74.41±5.70 |
| R1 | 118.93° | 37.27° | na | 3.82 | na | 0.59±0.07 | 138.75±11.7 |
| R2 | 119.16° | 37.76° | na | 5.33 | 3.83 | 0.00±0.00 | 133.9±10.31 |
| R3 | 119.56° | 37.05° | na | 0.80 | na | 1.34±0.16 | 51.36±6.72 |
| R4 | 119.49° | 37.11° | na | 2.31 | na | 4.88±0.59 | 174.76±9.75 |
| R5 | 119.24° | 37.07° | na | 25.70 | na | 21.74±2.61 | 214.50±9.20 |
| R6 | 119.18° | 37.11° | na | 14.38 | na | 3.48±0.42 | 92.70±5.63 |
| C1 | 120.30 | 37.67 | 0 | 27.19 | 29.74 | 1.19±0.15 | 38.06±2.77 |
| C2 | 119.98 | 37.42 | 0 | 27.50 | 28.41 | 1.00±0.12 | 36.69±6.18 |
| C3 | 119.91 | 37.30 | 0 | 27.16 | 27.06 | 1.58±0.19 | 89.62±3.09 |
| C4 | 119.63 | 37.11 | 0 | 24.67 | 28.07 | 2.21±0.27 | 91.96±4.71 |
| C5 | 119.47 | 37.12 | 0 | 23.45 | 28.01 | 2.09±0.25 | 60.04±4.06 |
| C6 | 119.30 | 37.12 | 0 | 22.98 | 27.67 | 2.86±0.44 | 85.57±3.41 |
| C7 | 119.06 | 37.24 | 0 | 22.17 | 27.48 | 3.57±0.43 | 91.60±3.42 |
| C8 | 118.98 | 37.29 | 0 | 21.84 | 27.19 | 4.68±0.56 | 94.66±3.58 |
| C9 | 118.94 | 37.35 | 0 | 21.48 | 27.30 | 4.01±0.48 | 110.22±4.88 |
| C10 | 118.93 | 37.40 | 0 | 22.41 | 27.52 | 4.01±0.48 | 110.21±4.89 |
| C11 | 118.92 | 37.52 | 0 | 23.78 | 27.54 | 3.11±0.38 | 114.57±6.18 |
| C12 | 119.02 | 37.67 | 0 | 24.34 | 27.50 | 2.84±0.34 | 107.35±6.50 |
| C13 | 119.15 | 37.72 | 0 | 24.41 | 27.72 | 2.54±0.30 | 91.57±6.18 |
| C14 | 119.23 | 37.74 | 0 | 24.13 | 27.34 | 2.90±0.35 | 97.00±6.17 |

Stations’ names that begin with “S”, “G”, and “R” refer to stations where seawater samples, groundwater samples and river water samples were collected, respectively. “C” means a triangle node on the coastline where no measurements were taken.The coordinates were obtained by a GPS handset with the WGS-84 reference system.

“na” means no values or not available in the study.

^a^Denotes seawater samples used to determine the background activity of Ra.

^b^Denotes groundwater samples used to determine groundwater end-member values of ^226^Ra.

**Table S2** ^226^Ra activities, river fluxes, suspended particles concentrations (SPC) and salinities in river water samples (2012).

| River | Salinity | Fluxes (m^3^/d)^*^ | SPC (kg/d)^*^ | ^226^Ra (dpm/100L) |
| --- | --- | --- | --- | --- |
| Yellow River | 5.3 | 1.61×10^8^ | 3.54×10^8^ | 133.9±10.31 |
| Jiaolai River | 0.8 | 6.91×10^5^ | 1.67×10^5^ | 51.36±6.72 |
| Xiaoqing River | 3.8 | 1.12×10^6^ | na | 138.75±11.70 |
| Wei River | 2.3 | 8.64×10^5^ | na | 174.76±9.75 |
| Yu River | 25.7 | 1.81×10^4^ | na | 214.5±9.70 |
| Bailang River | 14.4 | na | na | 92.70±5.63 |

*Data were obtained from Yellow River Water Resources Bulletin published by Yellow River Conservancy Commission of the Ministry of Water Resources of China.

“na” means that the water flux or suspended particles supplied by rivers to the Bay are negligible.

**Table S3** Parameters and calculations for water and salt mass balance models.

| Parameters | Values (2012) | Values (2014) |
| --- | --- | --- |
| Open-sea seawater salinity ($s_{s}$) | 27.34 | 29.67 |
| Freshwater salinity ($s_{f}$) | 0 | 0 |
| Water volume in the Bay ($V_{Bay}$) | 4.72×10^10^ m^3^ | 4.38×10^10^ m^3^ |
| Total salt mass ($M_{s}$) | 1.22×10^12^ m^3^psu | 1.24×10^12^ m^3^psu |
| Flushing time ($T_{f}$) | 36.6 d | 36.6 d |
| The Yellow River flux ($Q_{Y}$) | 1.61×10^8^ m^3^/d | 3.22×10^7^ m^3^/d |
| Total flux of other rivers ($\sum Q_{r,i}$) | 2.70×10^6^ m^3^/d | Not available |
| Precipitation ($P_{T}$) | 2.14×10^7^ m^3^/d | 2.19×10^6^ m^3^/d |
| Evaporation ($E_{T}$) | 1.34×10^7^ m^3^/d | 3.49×10^6^ m^3^/d |
| Open-sea seawater volume ($V_{s}$) | 4.45×10^10^ m^3^ | 4.18×10^10^ m^3^ |
| Freshwater volume ($V_{f}$) | 2.72×10^9^ m^3^ | 2.04×10^9^ m^3^ |
| Open-sea seawater flux ($Q_{s}$) | 1.21×10^9^ m^3^/d | 1.14×10^9^ m^3^/d |
| Freshwater flux ($Q_{f}$) | 7.43×10^7^ m^3^/d | 5.56×10^7^ m^3^/d |

**Table S4** Parameters and calculations for the ^226^Ra mass balance model.

| Parameters | Values |
| --- | --- |
| Mean ^226^*Ra* in groundwater (${}^{226}Ra_{gw}$) | 68.08 dpm/100L |
| Background value of ^226^Ra (${}^{226}Ra_{bg}$) | 21.89 dpm/100L |
| The coefficient of desorption of ^226^Ra ($\alpha_{des}$) | 600 dpm/kg |
| Mixing ^226^*Ra* flux ($(V_{Bay}{}^{226}Ra_{Bay}-V_{Bay}{}^{226}Ra_{bg})/T_{f}$) | 4.26×10^11^ dpm/d |
| Total ^226^*Ra* flux from the Yellow River ($^{226} Ra_{TYL}$) | 4.28×10^11^ dpm/d |
| Total ^226^*Ra* flux from other rivers ($^{226} Ra_{Tr}$) | 3.46×10^9^ dpm/d |

1. Corresponding author, email: hailongli@cugb.edu.cn [↑](#footnote-ref-2)
